# A neutrophil extracellular traps-related classification predicts prognosis and response to immunotherapy in colon cancer

**DOI:** 10.1038/s41598-023-45558-6

**Published:** 2023-11-07

**Authors:** Cheng Feng, Yuan Li, Yi Tai, Weili Zhang, Hao Wang, Shaopu Lian, E-er-man-bie-ke Jin-si-han, Yuanyuan Liu, Xinghui Li, Qifeng Chen, Meng He, Zhenhai Lu

**Affiliations:** 1 Department of Colorectal Surgery, Sun Yat-sen University Cancer Center, Sun Yat-sen University, Guangzhou, 510515 Guangdong China; 2 Department of Radiation Oncology, National Cancer Center/National Clinical Research Center for Cancer/Cancer Hospital and Shenzhen Hospital, Chinese Academy of Medical Sciences and Peking Union Medical College, Shenzhen, 518116 Guangdong China; 3 Department of Minimally Invasive Interventional Therapy, Liver Cancer Study and Service Group, Sun Yat-Sen University Cancer Center, Guangzhou, 510515 Guangdong China; 4 Department of Musculoskeletal Oncology, Sun Yat-Senen University Cancer Center, Guangzhou, 510515 Guangdong China; 5 Department of Radiation Oncology, The First Hospital of Hunan University of Chinese Medicine, Changsha, 410021 Hunan China; 6 Department of Cardiology General Hospital of Xinjiang Military Command, No. 359 Youhao North Road, Saybak District, Urumqi, 830001 Xinjiang China

**Keywords:** Cancer microenvironment, Chronic inflammation, Immunology, Oncology

## Abstract

Neutrophil extracellular traps (NETs) have been categorized as a form of inflammatory cell death mode of neutrophils (NETosis) involved in natural immunity and the regulation of adaptive immunity. More and more studies revealed the ability of NETs to reshape the tumor immune microenvironment (TIME) by limiting antitumor effector cells, which may impair the efficacy of immunotherapy. To explore whether NETs-related genes make vital impacts on Colon carcinoma (COAD), we have carried out a systematic analysis and showed several findings in the present work. First, we obtained the patient's data from The Cancer Genome Atlas (TCGA) and Gene Expression Omnibus (GEO) dataset, aiming to detect two NETs-associated subtypes by consensus clustering. For the purpose of annotating the roles of NETs-related pathways, gene ontology enrichment analyses were adopted. Next, we constructed a 6 novel NETs-related genes score using the Least Absolute Shrinkage and Selection Operator (LASSO) Cox regression model. We found that the NETs risk score was notably upregulated in COAD patient samples, and its levels were notably correlated with tumor clinicopathological and immune traits. Then, according to NETs-associated molecular subtypes and the risk signature, this study compared immune cell infiltration calculated through the estimate, CIBERSORT, TIMER, ssGSEA algorithms, tumor immune dysfunction, as well as exclusion (TIDE). Furthermore, we confirm that MPO(myeloperoxidase) was significantly upregulated in COAD patient samples, and its levels were significantly linked to tumor malignancy and clinic outcome. Moreover, multiplex immunohistochemistry (mIHC) spatial analysis confirmed that MPO was closely related to Treg and PD-1 + Treg in spatial location which suggested MPO may paly an important role in TIME formation. Altogether, the obtained results indicated that a six NETs-related genes prognostic signature was conducive to estimating the prognosis and response of chemo-/immuno-therapy of COAD patients.

## Introduction

Colon carcinoma (COAD) refers to a prevalent disease globally with high incidence and mortality rates^1^. Additionally, the incidence and mortality of COAD are elevating globally, with an estimated 60% increase by 2030^2^. Immunotherapy has appeared to be a promising therapy for COAD, and the rapid development in this field has made it a research hotspot. However, the efficacy of immune checkpoint inhibitors (ICIs) is predicted on the basis of the status of mismatch repair (MMR) status^3^. Recent research has indicated that immune status varies among deficient mismatch repair/microsatellite instability-high (dMMR/MSI-H) COAD, and more than 50% of patients still experience resistance to ICIs with an unknown mechanism^4^. The complexity of the tumor immune microenvironment (TIME) and the cancer inner-heterogeneity of the tumor contribute to the resistance to immunotherapy for COAD patients^5,6^. Meanwhile, with the advancement of technology, we have understood the complexity and diversity of the immune context of the tumor microenvironment(TME) as well as the impact on response to therapy^7,8^. As a result, detecting new biological markers concerning the therapeutic benefit of immunotherapy could help patients undergoing COAD receive more individualized therapies.

Inflammation is probably associated with cancer immunotherapy sensitivity, and some special subtypes of myeloid cells often contribute to nonresponsive of ICIs therapy^9–11^. Neutrophils are a vital component of circuits orchestrating the activation, orientation, and regulation of adaptive immune responses and chronic inflammation^12^. They exhibit the following immune functions, containing phagocytosis, reactive oxygen species (ROS) production and degranulation, as well as the formation and the release of sticky web-like structures consisting of decondensed chromatin filaments, known as NETs^13^. NETs, which are a network structure composed of DNA histones and proteins released by activated neutrophils, make a strong impact on immune status of the TME^14^. Therefore, more attention has been paid to the function of neutrophils and NETs in forming TIME^15^. Recent studies have indicated that NETs can stimulate tumor recurrence, metastasis, as well as the therapeutic resistance^16,17^. Moreover, anti-NETs can have synergistic antitumor effects in conjunction with immunotherapy,while the role of NETs in COAD immunotherapy remains unclear ^18–20^. As a result, it is necessary to detect and confirm NET and NETosis biomarkers for predictive/prognostic roles in COAD patients receiving immunotherapy.

At present, there is no reliable model for predicting the response of COAD to immunotherapy on the basis of NETs-related genes. Therefore, developing a model that categorizes patients in accordance with their response to immunotherapy using NETs-related genes would be highly beneficial. The objective of the present study is to detect NETs-related biomarkers and develop a NETs risk model which can predict the immune microenvironment, prognosis, as well as the response to chemotherapy and immunotherapy in COAD patients.

## Material and methods

### Data sets

The datasets analysed during the current study are available in the TCGA and GEO repository.Regarding the training set creation, the RNA-seq transcriptome profiles of 514 patients with COAD were acquired from the Cancer Genome Atlas (TCGA) (https://portal.gdc.cancer.gov/), as well as matching clinicopathological information. Meanwhile, a total of 585 patients were obtained from the Gene Expression Omnibus (GEO; accession No.: GSE39582; https://ncbi.nlm.nih.gov/geo/query/acc.cgi?acc=GSE39582) to construct the validation set. To further validate, we conducted a retrospective analysis of 66 patients diagnosed with IIIA-IIIB stage CRC who underwent primary radical operation treatment at Sun Yat-sen University Cancer Center (SYSUCC) between April 2006 and July 2011, with follow-up censoring on March 17, 2020.The Ethical Review Committee of Sun Yat-sen University Cancer Center approved the study, the ethics approval number is B2023-188-01, and informed consent was obtained from all patients. The study was conducted in accordance with the principles of the Declaration of Helsinki.

### NETs initial biomarkers

To identify initial biomarkers for NETs, we analyzed neutrophils and NETosis-related gene sets that were identified in previous research. Yi Zhang et al.^21^ has priorly summarized the assessment of NETs-associated genes herein. Briefly speaking, the gene set of neutrophils and that associated with NETosis was a summary of advances in NETs research in diverse diseases and immunity^13,22^.

### Consensus clustering

Using the R's Consensus Cluster Plus program, consensus clustering was implemented, and the NETs-associated molecular subtypes were identified. Ideal cluster numbers were evaluated from k = 2 to 10, and the process was subjected to 1,000 repetitions, and thus the results could be stable. Cluster map depiction was accomplished via the heatmap technique in R.

### Identification of differentially expressed genes (DEGs)

Via R's Limma ver. 3.40.2, differential expression of mRNAs was evaluated. The adjusted P values were checked for the false-positive TCGA data rectification. Besides, the mRNAs were considered expressed differentially when the | fold change| exceeded 2, and the adjusted P was below 0.05.

### Functional enrichment analysis

This study performed Kyoto Encyclopedia of Genes and Genomes (KEGG) and Gene Ontology (GO) assessments. As a result, the differential pathways and biological effects could be compared among the low vs. high NETs cohorts. For the KEGG and GO pathway evaluation, we employed R's "clusterProfiler" program^23^. The threshold settings for the foregoing enrichment assessments were p- and q-values of below 0.05.

### Gene set enrichment analysis (GSEA)

This study conducted GSEA to evaluate significant variations in the set of genes which were denoted between the NETs low and high cohorts in the enrichment of the MSigDB Collection (c2.cp.kegg.v7.4.symbols.gmt). Based on GSEA software (http://www.broadinstitute.org/gsea/index.jsp), we accomplished the analysis in this study.

### Characterization of immune landscape

We utilized CIBERSORT (https://cibersort.stanford.edu/) to identify immune characteristics between two NETs subgroups. We loaded their expression data from 1099 COAD samples into CIBERSORT and repeated the process 1,000 times, so that the relative proportions of 22 kinds of immunocytes could be estimated^24^. Subsequently, the estimated relative proportions were compared between the two NETs cohorts and a landscape map was exploited to report the results.

### Prediction of response to immunotherapy

By employing the Tumor Immune Dysfunction and Exclusion (TIDE) technique (http://tide.dfci.harvard.edu/), response to the immunotherapy was evaluated, which is capable of forecasting immunotherapy response based on two chief mechanisms for tumor immune escape: infiltration and dysfunction of T cells suppressed in CTL-low tumors.

### Somatic mutation analysis

Somatic mutation profiles of COAD samples were acquired from the TCGA GDC Data Portal in "maf" format. With the aid of R's "Maftools" package, the waterfall curves were plotted, thereby achieving the mutated gene visualization and summarization.

### Survival analysis

For the overall survival (OS) contrast among the low vs. high NETs risk populations, R's survival and survminer were exploited to perform the Kaplan–Meier (KM) assessment. We identified prospective prognostic indicators using Univariate Cox analysis, and identified the probability of risk score as an independent OS predictor in COAD through multivariate Cox regression.

### Construction of NETs-related risk signature

The NETs-related genes exhibiting a statistical significance in the univariate Cox procedure were subject to LASSO-based Cox analysis, thereby estimating the precise coefficient values for every identified relation. As a popular approach of regression analysis, LASSO enhances the predictive efficiency and interpretability of the resultant model through variable selection in conjunction with regularization.

### Retrospective patient cohorts and tissue microarrays multiplex immunohistochemistry (mIHC) analysis

We retrieved collected formalin-fixed, paraffin-embedded tumor samples from 66 IIIA-IIIB stage CRC patients collected at Tumor Bio-bank of Sun Yat-sen University Cancer Centre, provided in a tissue microarray (TMA) format. Further more, mIHC/IF was performed using an Opal Multiplex fIHC kit (PerkinElmer, Inc, Waltham, Massachusetts, USA).Tissue sections (4 µm thick) were labeled with primary antibodies against MPO(R&D,AF3667),CD8(ZSGB-BIO,ZA-0508), FOXP3(Abcam, 20,034) and PD-1(ZSGB-BIO,ZM-0381), followed by appropriate secondary antibodies.This was followed by the application of a fluorophore-conjugated tyramide signal amplification buffer (PerkinElmer, Inc) and the nuclear counterstain DAPI. A Vectra three pathology imaging system microscope (PerkinElmer, Inc) was used to obtain images, and these were analyzed using inForm software (V.2.4.2; PerkinElmer, Inc) ^25,26^ nd HALO TM (Indica Labs, Albuquerqe, New Mexico, USA)^27^.

### Statistical analysis

All data were analyzed using R software (v4.1.1); a P-value less than 0.05 was considered statistically significant. The “limma”R package was used to perform a difference analysis. The Wilcoxon test was used for data that did not accord with a normal distribution. A t-test was used for normally distributed data. Kaplan–Meier method were used to assess the prognostic value of DEGs. The heatmaps were performed via the R “pheatmap” package.Univariate and multivariate Cox regression models (both *P* < 0.05) were conducted to examine the independence of the clinical NETs-related risk signature.

## Results

### Identification of two NETs-associated subtypes and correlation analysis through consensus clustering 

**Figure 1:**
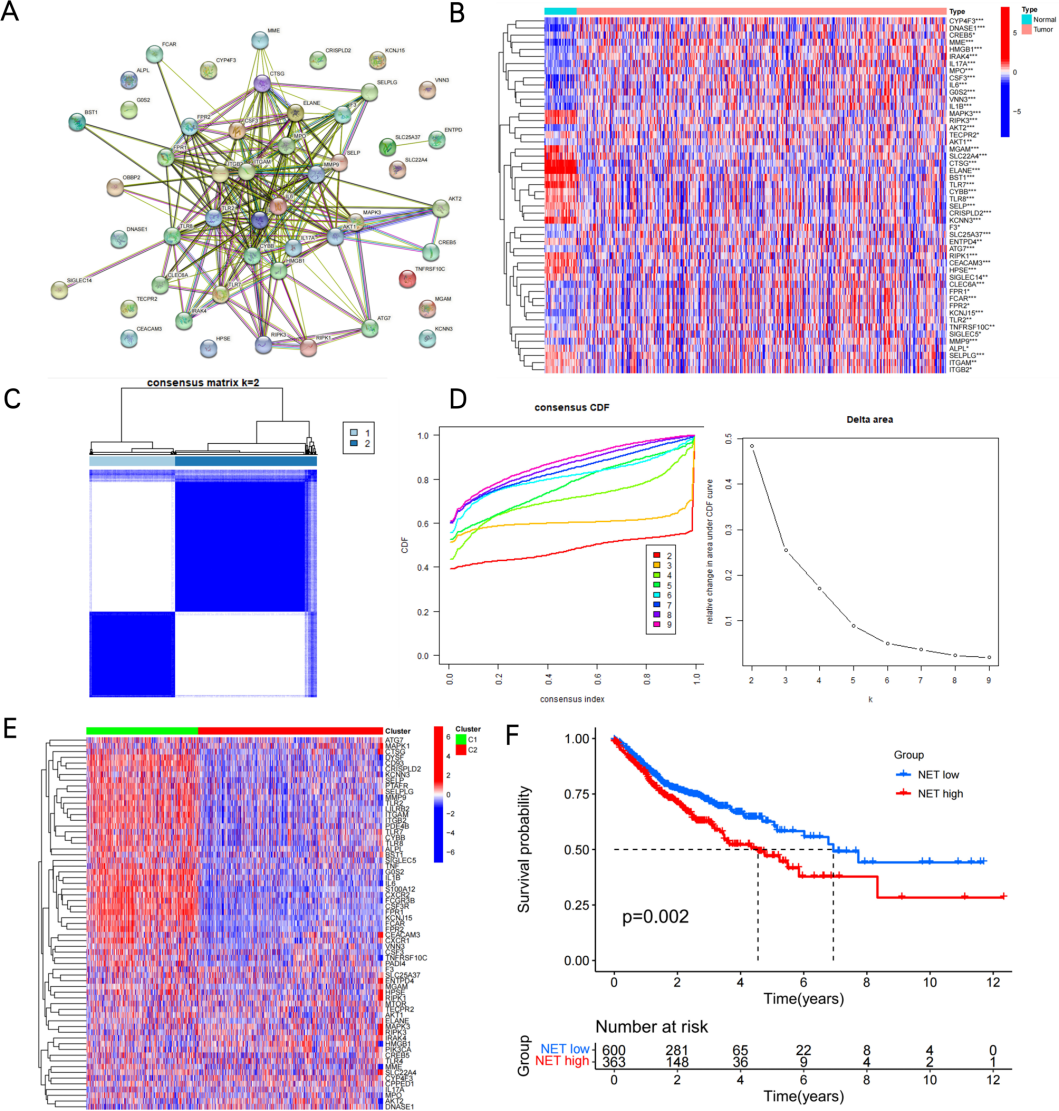
Identification of NETs-associated subtypes using consensus clustering. (**A**) Protein–protein interactions among the NETs-associated genes are depicted. (**B**) A heatmap illustrates the 50 NETs gene expression profiles among normal and COAD samples in the TCGA database. (**C**) Another heatmap displays the consensus clustering solution (k = 2) for 69 genes in COAD samples. (**D**) The delta area curve of consensus clustering shows the relative change in areas under the cumulative distribution function (CDF) curve for k = 2 to 10. (**E**) The heatmap of 69 NETs-related gene expressions in different subtypes is shown, with red representing high expression and blue representing low expression. (**F**) The Kaplan–Meier curves of OS in NETs-high and NETs-low subtypes are presented. *P < 0.05, **P < 0.01, ***P < 0.001, and ****P < 0.0001.

Previous research has used 69 genes to be the NETs-initial biomarkers^21^. We detected a total of 50 NETs-associated genes to be the initial biomarkers for signature training and carried out a protein–protein interaction (PPI) network analysis of these genes on the basis of the STRING database (Fig. 1A). This study explored the expression patterns of NETs-related genes in normal and COAD samples, discovering that 29 are upregulated genes and 21 are downregulated genes with a false discovery rate < 0.05 (Fig. 1B, Supplementary Table 1). Next, we identified the NETs-associated clusters of COAD using consensus clustering, which revealed two distinct NETs gene expression patterns (clusters 1, C1 and clusters 2, C2) with good internal consistency and stability (Fig. 1C,D). The expression profile of NETs between the C1 and C2 groups is shown in Fig. 1E. A total of 600 patients in C1 were defined as the NETs-low subtype and 363 patients in C2 as the NETs-high subtype, with survival analyses showing that these subtypes had different clinical outcomes (Fig. 1F).

### Identification of differential NETs-related genes and signal pathway analysis

**Figure 2:**
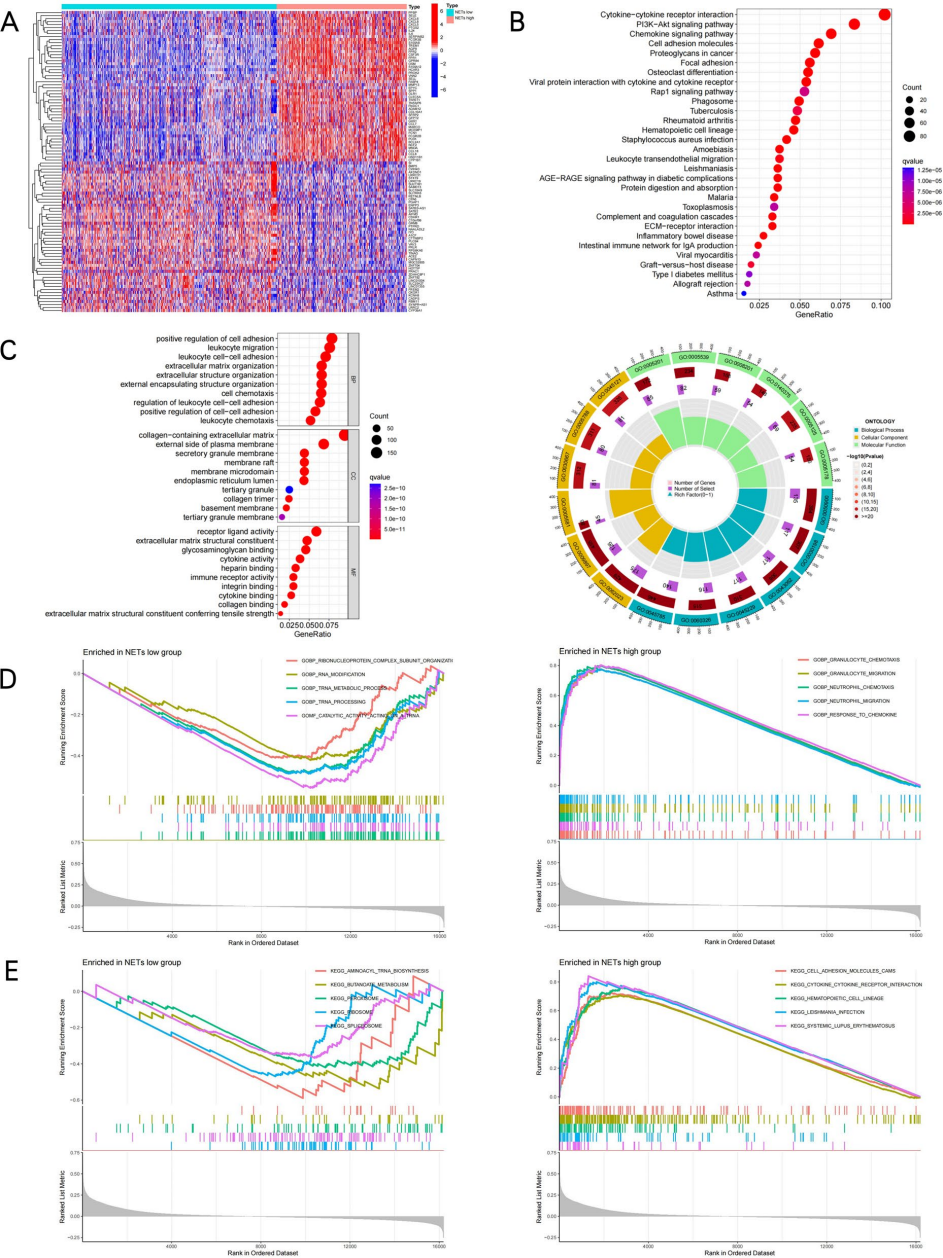
Identification of differently expressed genes (DEGs) and underlying signal pathways in different subtypes. (**A**) The heatmap visually displays the distribution of DEG expression between NETs-high and NETs-low subtypes. (**B**) Dots plot presents the KEGG signaling pathway enrichment analysis. (**C**) Dots plot and Circle diagram presents the GO signaling pathway enrichment analysis belonged to BP, CC, and MF for DE-IRGs.(Yellow, green, and blue represents Cellular Component, Molecular Function, and Biological Process, respectively.) (**D**) Set of genes enriched in NETs groups:GSEA analysis was performed on GOBP(E), KEGG(F) datasets (*P* < 0.05).

As the NETs-low subtype was related to favorable clinical findings and the NETs-high subtype was related to dismal prognosis, this study detected differentially expressed genes (DEGs) and signal pathways in each subtype, aiming to understand the molecular mechanisms underlying the modulation of prognosis. The present work compared the distribution of DEG expression between the NETs-high and NETs-low subtypes (Fig. 2A) and identified the top 30 KEGG pathways (Fig. 2B), and 30 different GO terms engaged in biological process (BP), cellular component (CC), and molecular function (MF) (Fig. 2C). According to functional enrichment analysis, the most associated signaling pathway for NETs is referred to as "Cytokine-cytokine receptor interaction," with the most enriched terms in BP, CC, and MF being "positive regulation of cell adhesion," "receptor ligand activity," and "collagen-containing extracellular matrix," respectively. These results suggested that the NETs-high subtype was correlated with the inflammatory response microenvironment and pro-tumor metastasis. For better identifying related pathways of the NETs-high subgroup, GSEA was carried out, which found that granulocyte chemotaxis migration and cell adhesion molecules CAMs pathways were enriched in this subgroup, whereas tRNA metabolisms like ribonucleoprotein complex subunit organization and aminoacyl tRNA biosynthesis and tRNA metabolism process was enriched in NETs-low subgroup (*P* < 0.05; Fig. 2D,E).

### Somatic mutations in NETs-high and NETs-low subtypes 

**Figure 3:**
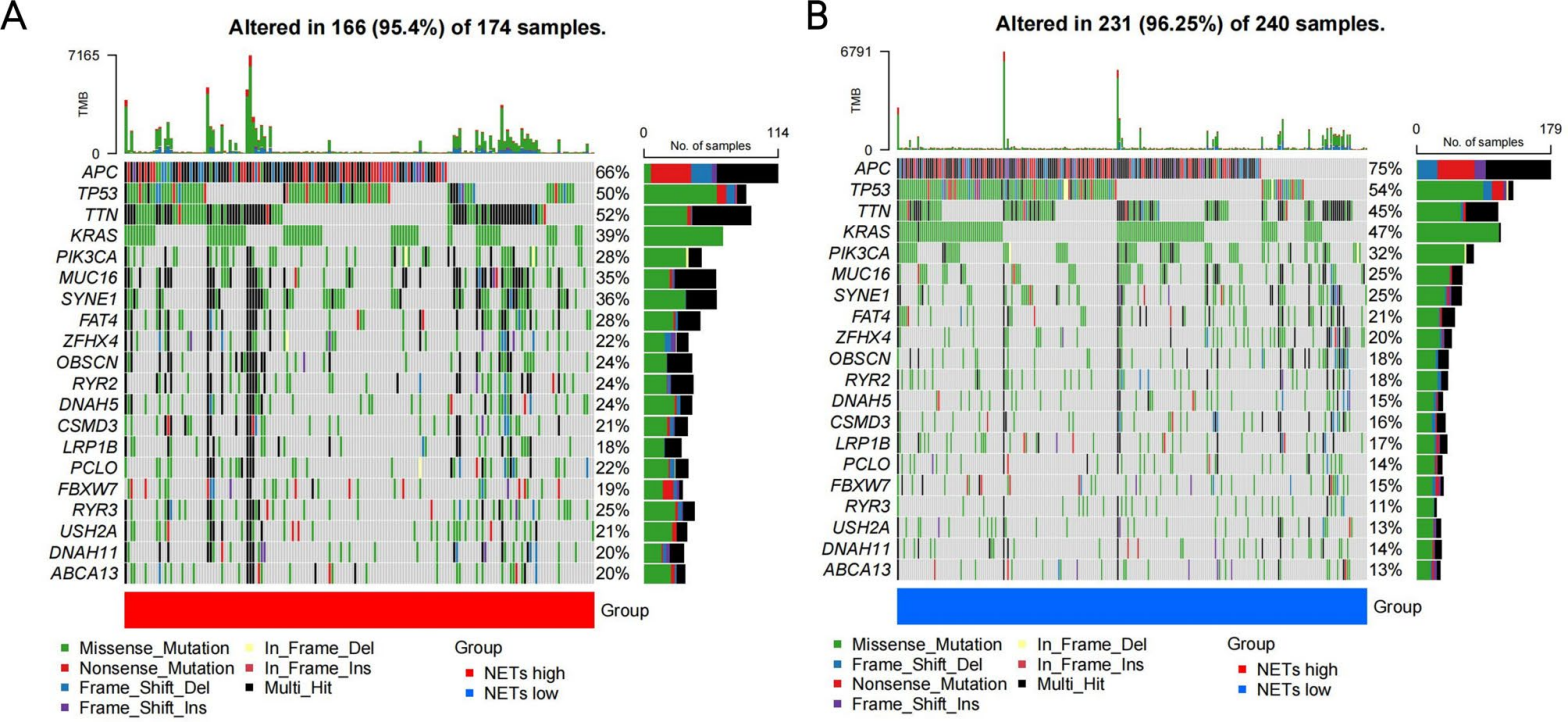
A comparison of somatic mutations in different NETs subtypes, with visualizations of the top ten most frequently mutated genes in the NETshigh (**A**) and NETs-low (**B**) subtypes.

This study identified distinct somatic mutation profiles in the NETs-high and NETs-low subtypes in order to gain further understanding of the immunological nature of the NETs subpopulation. Although APC, TP53, TTN, KRAS, PIK3CA, MUC16, and SYNE1 were regarded as the most frequently mutated genes, the relative frequencies varied between the subtypes. The top five genes which had the highest mutation frequencies in the high-NETs group were APC (66%), TTN (52%), TP53 (50%), KRAS (39%), as well as SYNE1 (36%), whereas the top five genes in the low-NETs group were APC (75%), TP53 (54%), KRAS (47%), TTN (45%), and PIK3CA (32%) (Fig. 3A,B).

### Tumor microenvironment landscape in NETs-high and NETs-low subtypes

**Figure 4:**
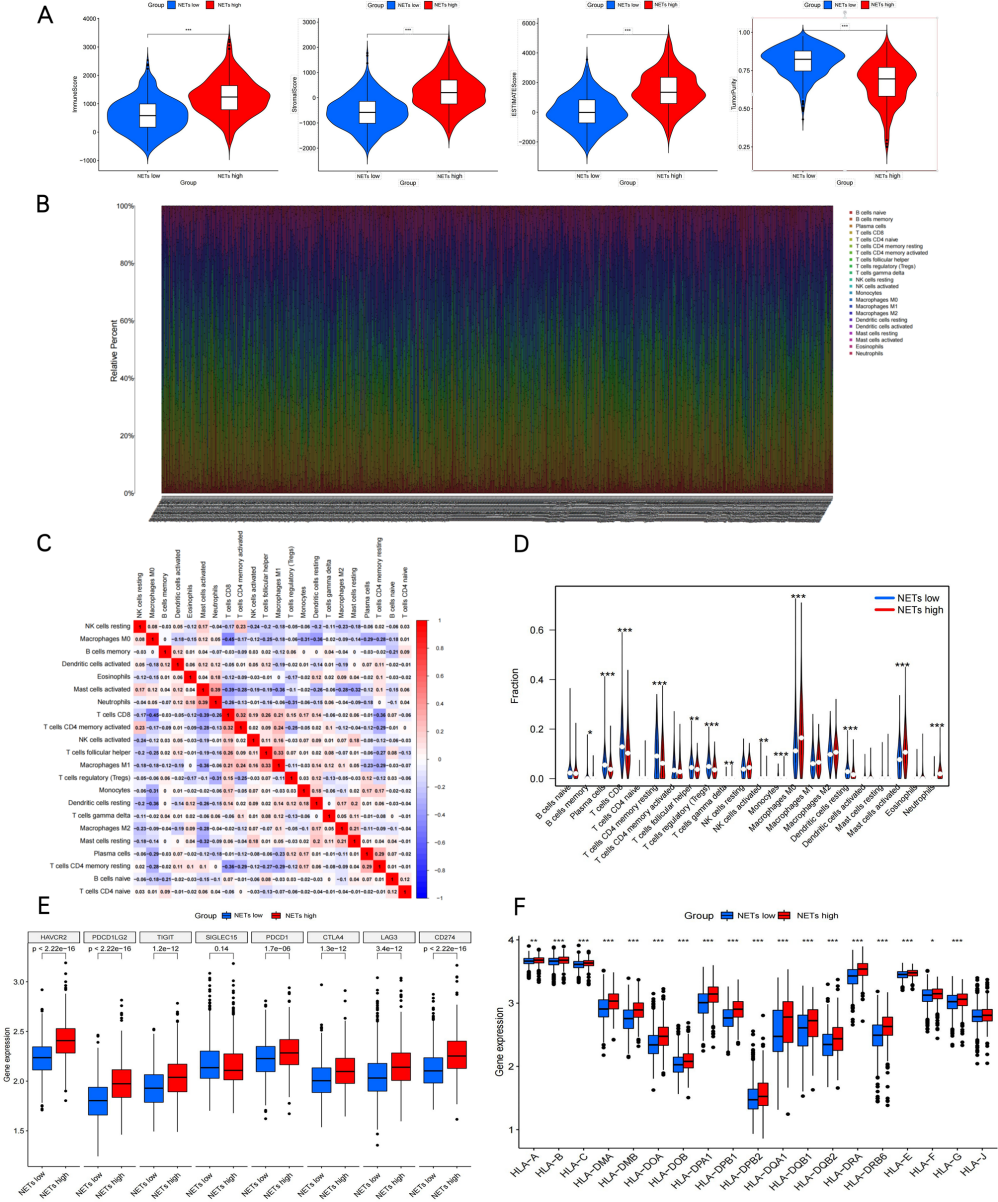
Immune landscape of NETs-high and NETs-low subtypes. (**A**) Violin plots show the median, and quartile estimations for each immune score, stromal score,ESTIMATE score, and tumor purity score. (**B**) Relative proportion of immune infifiltration in NETs-high and NETs-low subtypes. (**C**) Correlation analysis of each infifiltrating immune cells in the colon cancer; (**D**) Violin plot visualizes signifificantly different immune cells between different subtypes; Box plots present differential expression of multiple immune checkpoints (**E**), and HLA genes (**F**) between NETs-high and NETs-low subtypes. *P < 0.05, **P < 0.01, ***P < 0.001, and ****P < 0.0001.

In previous studies, TME and inflammatory response are found to have critical effects on cancer progression^28,29^. Besides, NETs are increasingly found to affect anticancer immunity activation significantly. The present work examined TME compositions in both subtypes to shed more lights on NETs states with regard to their immunological nature. Overall, NETs-high subtype had increased immune/stromal/ESTIMATE scores but decreased tumor purity in relation to the NETs-low subtype(Fig. 4A).Then, based on the CIBERSORT approach in conjunction with the LM22 signature matrix, this study evaluated the existing differences in immune infiltration of 22 types of immune cells between the two subtypes. Figure 4B shows the findings from 1099 patients in the TCGA and GEO. Besides, tumor-infiltrating immune cell interactions were detected, and the correlation analysis of each infiltrating immune cell in those COAD patients was conducted (Fig. 4C). Specifically, patients with the NETs-Low subtype showed significantly increased percentages of B cell plasma, B cell memory, T cells CD8, T cells CD4 memory resting, T cells follicular helper, T cells regulatory (Tregs), NK cells activated, as well as Dendritic cells resting. In addition, patients undergoing the NETs-High subtype revealed notably enhanced percentages of Monocytes, Macrophages M0, Mast cells activated, as well as Neutrophils (Fig. 4D).Furthermore, most of the human leukocyte antigen (HLA) genes and immune checkpoints (such as HAVCR2, PDCD1LG2, TIGIT,SIGLEC15,PDCD1,CTLA4,LAG3,CD274) presented upregulation in the NETs-high subtype, whereas the opposite trend was found in the NETs-low subtype (Fig. 4E,F). The obtained findings indicated that the NETs-high subtype is related to an immunosuppressed state phenotype, whereas the NETs-low subtype is associated with an immune-activated state phenotype.

### Construction and validation of the NETs-related risk signature 

**Figure 5:**
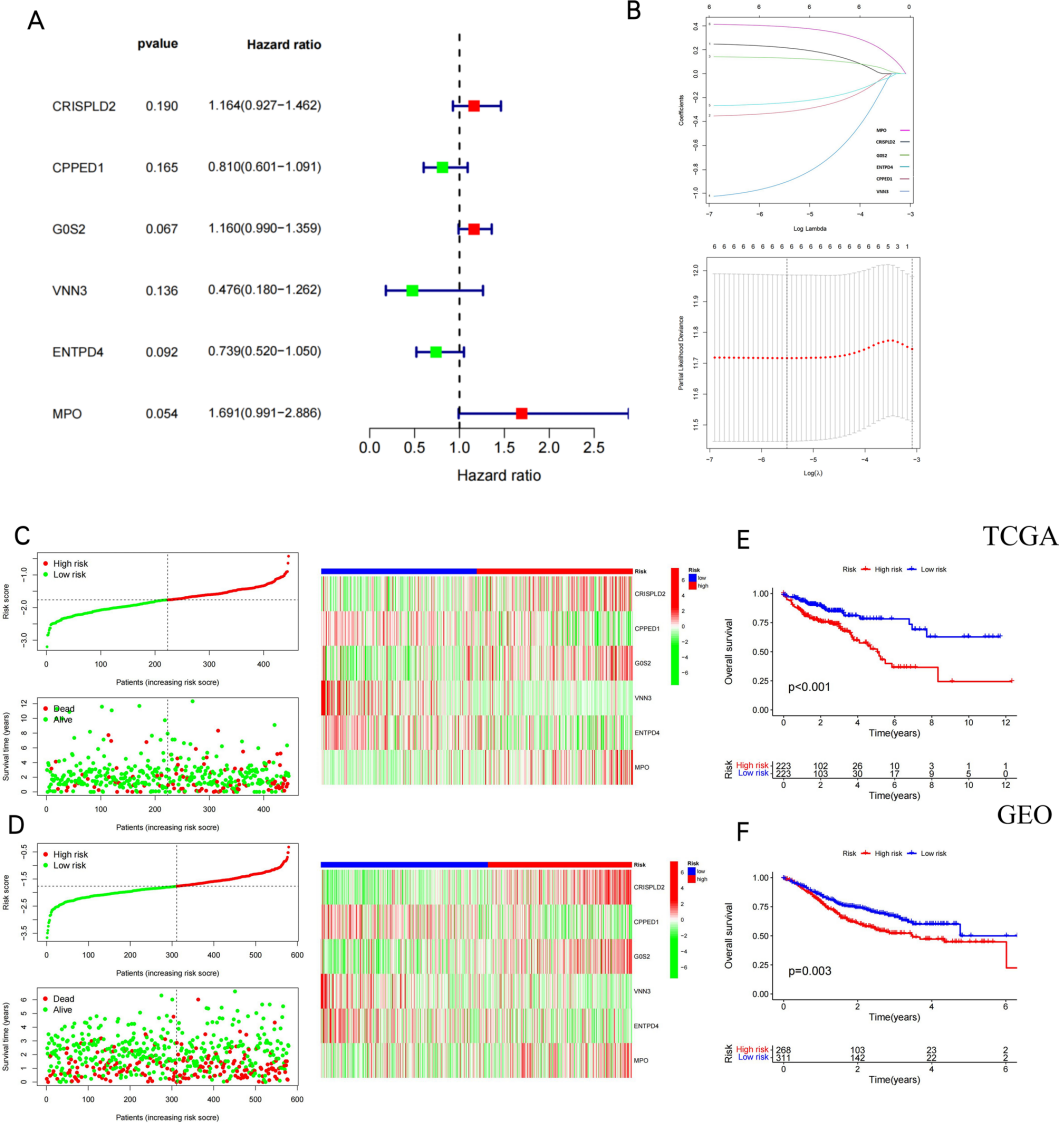
Construction and validation of the NETs risk signature. Risk score analysis, Kaplan–Meier analysis for the validation of prognostic model in TCGA set and GSE17538 set. (A) Univariate Cox analysis evaluates the prognostic value of the NETs genes in terms of overall survival. (**B**) Lasso Cox analysis identified the six genes most associated with OS in the TCGA dataset.Survival status and heatmap accompanied with the risk score in TCGA cohort (**C**) and GSE17538 cohort (**D**). Kaplan–Meier suggested that high-risk score group had shorter overall survival than low-risk score group in TCGA cohort (**E**) and GSE17538 cohort (**F**).

In this study, a prognostic model on the basis of NETs-related genes was created. Cox univariate analysis identified six NETs-related genes that were most strongly linked to patient overall survival (OS) (Fig. 5A). LASSO regression analysis was adopted for selecting six NETs-related genes for the prediction model (Fig. 5B). The risk-score model was developed using the following algorithm: Risk score = (0.2176)*CRISPLD2 + (− 0.3205)*CPPED1 + (0.1302)*G0S2 + (− 0.9115)*VNN3 + (− 0.2437)*ENTPD4 + (0.3926)*MPO. Additionally, the risk-score distribution of each patient showed a high correlation with each patient’s survival status in the TCGA cohort (Fig. 5C) and GSE17538 cohort (Fig. 5D) dataset. Based on KM analysis, we further identified the prognostic significance of this risk profile in COAD (Fig. 5E). As shown in the TCGA cohort, a high-risk score was discovered to be in line with poor OS. This was deeply verified through the comparable findings in the GSE17538 cohort (Fig. 5F).

### Estimation of NETs risk signature with tumor microenvironment

**Figure 6:**
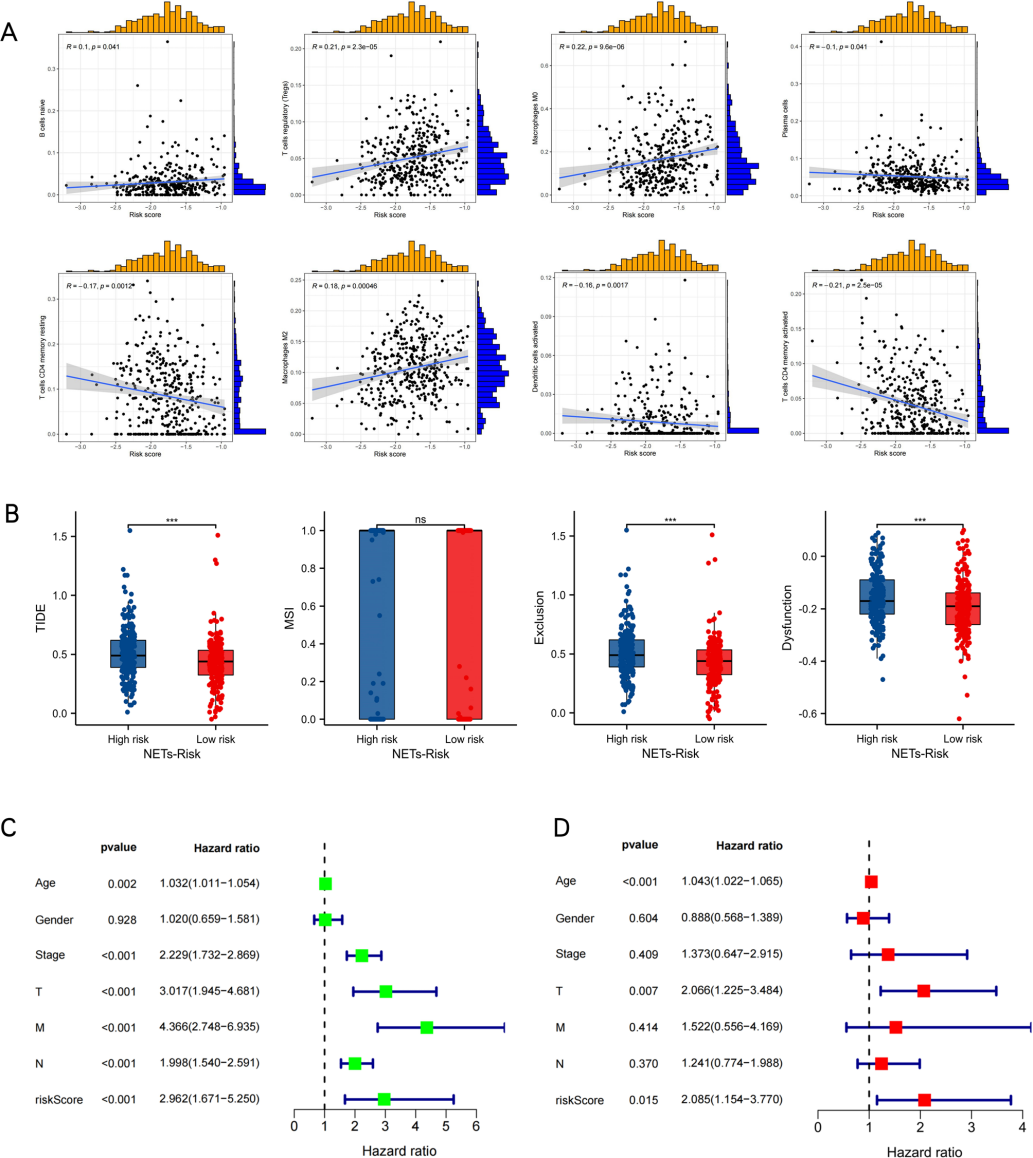
The correlation between the NETs risk signature and the tumor microenvironment. (**A**) Scatter plots show the relationship between the risk score and the infiltration of various immune cells, such as T cells regulatory (Tregs), B cells naive, Plasma cells, T cells CD4 memory resting, T cells CD4 memory activated, Macrophages M0, Macrophages M2, and Dendritic cells activated.Univariate (**B**) Box plots present the TIDE, MSI, and T cell exclusion and dysfunction score for different NETs-risk groups (**C**) and multivariate (**D**) Cox analyses evaluate the independent prognostic value of NETs risk signature in COAD patients.

Since NETs exert prominent biological roles in antitumor immunoreactions, a thorough investigation was made concerning the correlation of their risk score with the TME. As suggested by the results (Fig. 6A), patients with heightened risk scores displayed negative linkages to the resting of memory plasma and CD4 T cells, and activation of CD4 T and dendritic cells, while exhibiting positive linkages to the modulation of naive B, T cells, together with M0 and M2 macrophages. The higher the TIDE score, the stronger the immune evasion potential and, accordingly, the more probable the patients to benefit from the ICIs therapy^30^. The significance of NETs risk signature for forecasting the potential immunotherapy efficacy in the clinical setting was assessed by TIDE. It was found that compared to the high NETs risk population scoring high TIDE scores, a superior prognosis could perhaps be attained for the low NETs risk population scoring low TIDE scores. No inter-group difference was noted regarding the microsatellite instability (MSI) score. In contrast to the high NETs risk population, the T cell exclusion scores were lower and the T cell dysfunction was weaker among the low NETs risk population (Fig. 6B). The independent prognostic significance of NETs risk signature was evaluated through uni- and multivariate Cox regressions. As revealed by the univariate assessment, a high NETs risk score was linked significantly to an inferior OS (Fig. 6C). The multivariate results implied the possible usage of NETs risk score as an independent prognostic biomarker for individuals suffering from COAD (Fig. 6D).

### Evaluation of the relationship between the NETs risk signature and immune status in COAD

**Figure 7:**
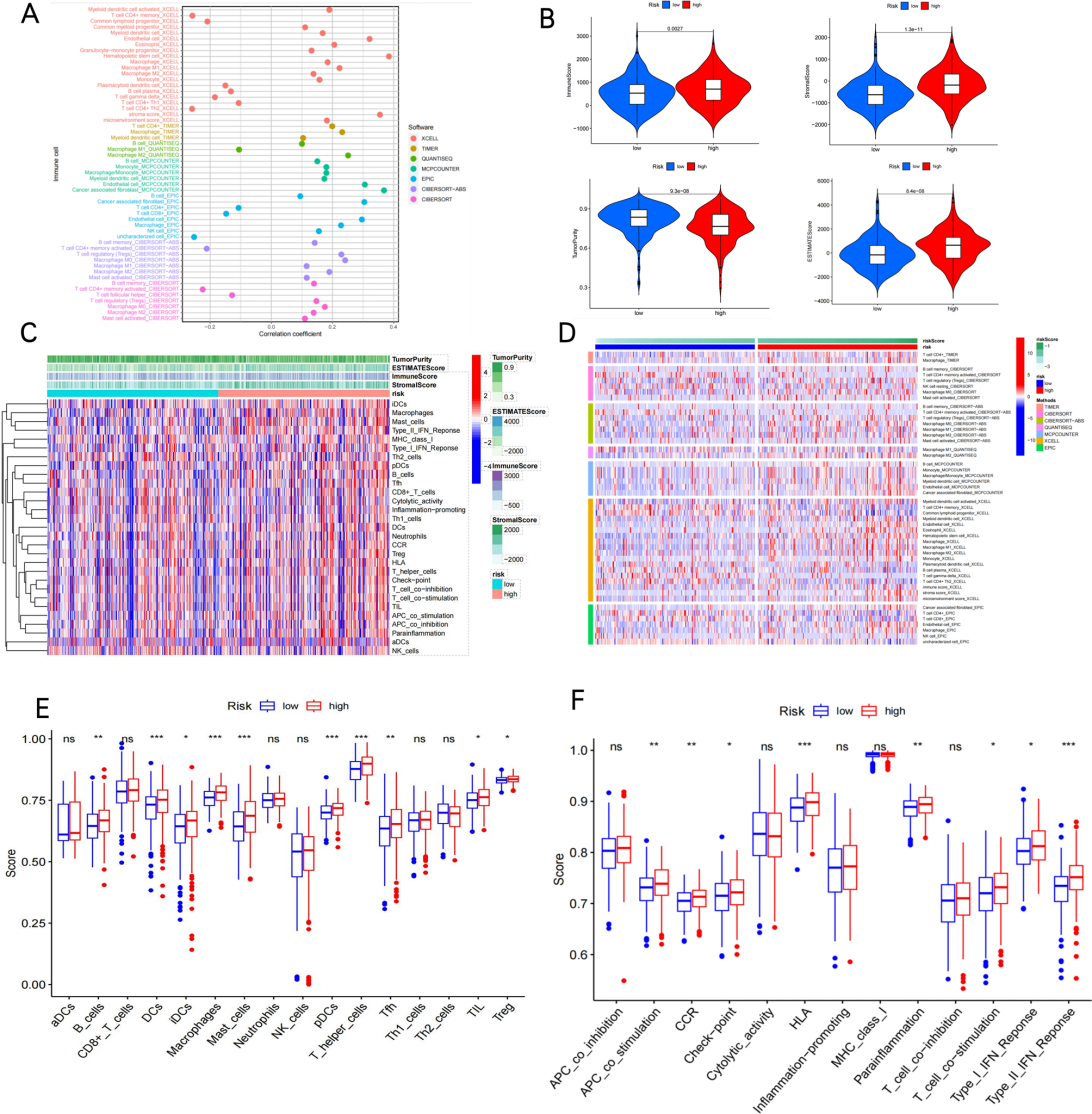
Immune landscape of Risk-high and Risk-low subtypes. (**A**) Immune cell Correlation coefficient analysised by seven software. (**B**) Violin plots show the immune scores, stromal scores,ESTIMATE scores, and tumor purity scores using ESTIMATE. (**C**) Heatmap of the association between the infifiltration immune cells and immune score, stromal score,ESTIMATE score, tumor purity score . (**D**)Heatmap of the immune cell infifiltration between the two clusters and analysised by seven methods.Box plots present differential expression of multiple infiltrating immune cells (**E**), and immune function (**F**) between Risk-high and Risk-low subtypes. *P < 0.05, **P < 0.01, ***P < 0.001, and ****P < 0.0001.

The immune infiltration in the TME makes an important impact on tumor progression and disease prognosis. Initial results indicate that the NETs risk model can determine the immunotherapy response of COAD and correlate with the infiltrating immune cells. Thus, we conducted further investigations into the immune landscape corresponding to the NETs risk model.Firstly, we employed various algorithms to quantify immune and stromal cells in the TME based on the transcription expression profile information, and we analyzed the connection between the risk score and immune and stromal cells. According to the obtained results, hematopoietic stem cell, stroma score, Cancer-associated fibroblast, and endothelial cell were significantly positively correlated, whereas T cell CD4 + memory, T cell CD4 + Th2, and T cell CD4 + memory activated were negatively correlated (Fig. 7A).The ESTIMATE algorithm examined the above-mentioned results and discovered that the immune score, stromal score, and ESTIMATE score were higher and that the tumor purity was lower in the high-risk subtype(Fig. 7B). Next, we analyzed the composition of the TME between high-risk and low-risk subtypes. There existed an association between the infiltration immune cells and immune score, stromal score, ESTIMATE score, and tumor purity score in the two risk groups, as shown in Fig. 7C.Moreover, the immune status of each patient in the low-risk group and high-risk group exhibited a different degree of heterogeneity in the heatmap (Fig. 7D). Accordingly, a variety of tumor-infiltrating immune cells showed a positive relationship to a higher risk score, suggesting the apparent effects of these cells on the pathogenesis of COAD (Fig. 7E). Similarly, the scores of immune-associated function pathways were notably higher in the high-risk group (Fig. 7F). Furthermore, this also conforms to the findings of tumor-infiltrating immune cells.

### MPO depicts the tumor immune microenvironment and prognosis of COAD

**Figure 8:**
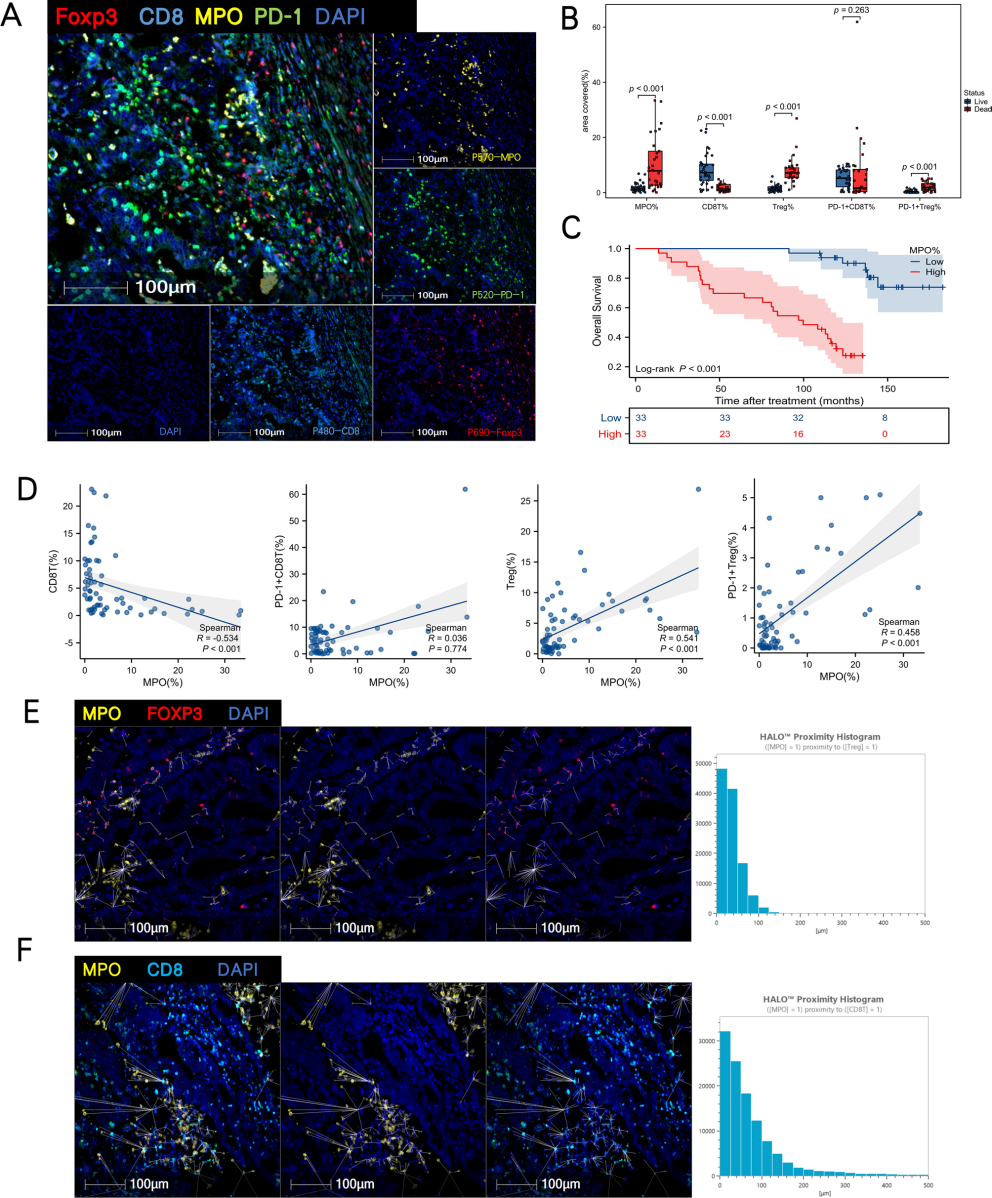
Retrospective patient cohorts and tissue microarrays multiplex immunohistochemistry (mIHC) analysis. (**A**) Immune landscape of TME analysed by multiplex immunohistochemistry (mIHC):marked by P690-Foxp3,P480-CD8T,P570-MPO,P520-PD-1,DAPI. (**B**) The expression of MPO(%), CD8T(%),Treg(%), PD-1 + CD8T(%), PD-1 + Treg(%) in different outcome patients. (**C**) Kaplan–Meier curves of OS in MPO-high and MPO-low subtypes among CRC patients from Sun Yat-sen University Cancer Centre. (**D**) Association of MPO(%) with CD8T(%), Treg(%), PD-1 + CD8T(%), PD-1 + Treg(%) in the TME. (**E**) Spatial analysis by HALO TM of MPO proximity to Treg in the TME. (F)Spatial analysis by HALO TM of MPO proximity to CD8T in the TME. *P < 0.05, **P < 0.01, ***P < 0.001, and ****P < 0.0001.

MPO is a kind of enzyme secreted by activated neutrophils and as an important component of NETs,which plays a crucial role in the tumor immune microenvironment^31^. As per previous results, MPO was found to be the most important gene in the 6-gene signature. To further investigate its significance, we collected clinical data from 66 IIIA-IIIB primary treatment COAD patients. The baseline characteristics of these patients are summarized in Table 1.We further examined the expression of MPO in COAD samples and their matched infiltrating immune cell:CD8T,Treg and their PD-1 expression (Fig. 8A).The results show that MPO,Treg and PD-1 + Treg were significantly expressed in the worse outcome samples as compared to their counterparts(Fig. 8B).Univariate analysis revealed that factors associated with inferior OS included MPO(%) (*P* = 0.009), CD8T(%) (*P* < 0.001) and Treg(%) (*P* = 0.015) (Table 2).Further Kaplan–Meier analysis implied that MPO level is reversely correlated with the survival of COAD patients using SYSUCC cohorts, showing that COAD patients with high a level of MPO had a worse prognosis (Fig. 8C).In addition, in order to better understand the immune infiltration in the TME, we used the Halo software for tissue microarray statistical analysis. The results showed a negative correlation between MPO and CD8T, and a positive correlation between Treg and PD-1 + Treg(Fig. 8D). The final spatial analysis further confirmed the close relationship between MPO and Treg in terms of spatial location, with the distribution distance more closely than CD8T(Fig. 8E,F).

**Table 1:** Patient characteristics at baseline (n = 66).

Characteristics	Overall (No.%)
Gender, n (%)	
Female	37 (56.1%)
Male	29 (43.9%)
Age, n (%)	
≤ 60	34 (51.5%)
> 60	32 (48.5%)
Primary site, n (%)	
Rectum	49 (74.2%)
Colon	17 (25.8%)
BMI, n (%)	
Abnormal	33 (50%)
Normal	33 (50%)
R0 resection, n (%)	
Yes	65 (98.5%)
No	1 (1.5%)
Max diameter of tumor(cm), median (IQR)	3.5 (3, 4.5)
Ratio of metastatic lymph nodes, median (IQR)	0.11438 (0.076923, 0.17402)
Pathological type, n (%)	
Middle differentiated carcinoma	57 (86.4%)
Signet-ring cell carcinoma	2 (3%)
Well differentiated carcinoma	3 (4.5%)
Poorly differentiated carcinoma	4 (6.1%)
Total retrieved lymph nodes, n (%)	
< 12	29 (43.9%)
≥ 12	37 (56.1%)
Number of metastatic lymph nodes, n (%)	
1	38 (57.6%)
2	20 (30.3%)
3	8 (12.1%)
Postoperative adjuvant chemotherapy, n (%)	
Yes	44 (66.7%)
No	22 (33.3%)
CEA(ng/ml), median (IQR)	2.27 (1.57, 6.215)
CA199(U/ml), median (IQR)	12.15 (7.155, 24.88)
MPO(%), median (IQR)	2.1245 (1.0935, 6.791)
CD8T (%), median (IQR)	3.1565 (1.113, 7.596)
Treg (%), median (IQR)	2.356 (0.9995, 6.324)
PD-1 + CD8T (%), median (IQR)	4.543 (0.93175, 8.3685)
PD-1 + Treg (%), median (IQR)	0.569 (0.08675, 1.4763)

**Table 2:** Univariate and multivariate analyses of prognostic factors for overall survival.

Characteristics	Univariate analysis		Multivariate analysis	
	Hazard ratio (95% CI)	*P* value	Hazard ratio (95% CI)	*P* value
Gender, Female vs. Male	1.510 (0.728–3.132)	0.269		
Age, ≤ 60 vs > 60	0.708 (0.341–1.471)	0.355		
BMI, Abnormal vs. Normal	1.347 (0.647–2.805)	0.427		
Pathological type		0.369		
Middle differentiated carcinoma	Reference			
Well differentiated carcinoma	1.819 (0.427–7.748)	0.418		
Poorly differentiated carcinoma	1.261 (0.296–5.370)	0.754		
Signet-ring cell carcinoma	0.000 (0.000–Inf)	0.997		
Total retrieved lymph nodes(n), < 12 vs ≥ 12	0.995 (0.474–2.085)	0.989		
Number of metastatic lymph nodes(n)		0.297		
1 vs. 3	2.772 (0.639–12.028)	0.173		
2 vs. 3	2.224 (0.479–10.318)	0.307		
Postoperative adjuvant chemotherapy, No vs. Yes	0.655 (0.312–1.374)	0.263		
MPO(%), ≤ 3.77 vs > 3.77	14.028 (5.721–34.401)	< 0.001	4.035 (1.416–11.499)	0.009
CD8T(%), ≤ 3.20 vs > 3.20	38.380 (8.665–170.002)	< 0.001	25.855 (3.854–173.432)	< 0.001
Treg(%), ≤ 3.12 vs > 3.12	88.834 (11.779–669.955)	< 0.001	19.334 (1.759–212.460)	0.015
PD-1 + CD8T(%), ≤ 2.11 vs > 2.11	2.537 (1.208–5.327)	0.014	0.747 (0.327–1.709)	0.490
PD-1 + Treg(%), ≤ 1.06 vs > 1.06	10.194 (4.448–23.365)	< 0.001	2.503 (0.903–6.937)	0.078
CEA(ng/ml), ≤ 5 vs > 5	0.832 (0.335–2.067)	0.693		
CA199(U/ml), ≤ 35 vs > 35	0.962 (0.332–2.783)	0.943		

## Discussion

Immunotherapy has became one of the most promising therapeutic techniques in COAD treatment^32^. However, only a subset of COAD patients benefit from ICIs treatment due to primary or acquired resistance and ICIs-related toxic side effects ^33^.To further improve the effectiveness of these treatment strategies, identifying factors that limit the benefit of these treatments is critical.It has shown that the type, functional localization, density, as well as location of innate and adaptive immune cells in the TME are essential for cancer evolution^10,34–36^.As the immune status of TME differ in individuals that highlight the need for identifying prognostic biomarkers for the application of immunotherapy for COAD patients ^37^. Neutrophils are the most abundant immune cells in the TME,and the activated neutrophils-released NETs have an essential effect on TIME formation, which suggests that NETs within TIME might be the candidate ICIs therapeutic target^14^. Due to the imbalance of polygenic expression regulation engaged in the incidence and development of tumors, it has been discovered that multigene score has better predictive efficacy.The current work detected a NETs-related prognostic risk model comprised of six genes that can predict clinical outcomes and ICIs treatment responses in COAD patients. High-risk patients exhibited lower objective response rates to ICIs therapy and earlier cancer progression, whereas low-risk patients had more treatment benefits and more prolonged survival in the TCGA and GEO cohorts.Furthermore, the findings of this study shed light on the role of NETs in the tumor microenvironment (TME) and demonstrate that, NETs can promote immunosuppression and undermine the effectiveness of immunotherapy under specific conditions.Consequently, our study can potentially enhance the accuracy of ICIs response and survival probability predictions for COAD patients.

The NETs-risk model is composed of six genes: CRISPLD2, CPPED1, G052, VNN3, ENTPD4, and MPO. CPPED1, known to be a negative regulator of Akt signaling, makes an impact on the innate immune system pathways and is associated with carcinogenesis in various cancers^38,39^. CRISPLD2 makes a vital impact on maintaining cell structure, which can participate in immune response, inhibit inflammation, or be involved in cell motility^40^.G0S2 has been identified as a tumor suppressor that opposes MYC activity^41^,which related to neutrophils, activated and resting mast cells, M0 and M1 macrophages, regulatory T cells (Tregs), resting dendritic cells, together with resting CD4 memory T cells^42^. VNN3 is a member of the vanin family that is engaged in oxidative stress and inflammation^43^,which has also been discovered to be engaged in the incidence and development of cancer ^44^. ENTPD4 participates in promoting gastric cancer development and prostate cancer progression^45,46^. As per previous results, MPO was found to be the most important gene in the 6-gene signature,which refers to one of the most abundant proteins in neutrophils and is preserved in azurophilic granules and released under the condition that neutrophils are facilitated. MPO results in DNA decondensation, binds to DNA and catalyzes oxidative reactions, which can promote the relocation of ELANE from the cytoplasm to the nucleus^47^. In addition, MPO plays an essential role in making NETs and promotes NETs formation cell-autonomously and is considered a representative marker of NETosis ^48^. Furthermore, MPO is involved in the ferroptosis of tumor neutrophils, causing immune suppression of TME in cancer ^49^. In particular, neutrophil-derived MPO may make a meaningful impact on cancer development and progression ^50^.Therefore, we utilized SYSUCC data to confirm that high MPO levels were associated with a poor prognosis for CRC. Subsequent mIHC analysis further demonstrated that MPO was positively correlated with Treg and negatively correlated with CD8T in the TME.To minimize the introduction of batch effects and bias, we ensure a consistent and continuous patient selection process, along with a consistent baseline level. The data obtained from public datasets TCGA and GEO normalized to further reduce any potential deviations.

Studies have shown that the dMMR share a very low proportion in CRC ,and even more than half still experience resistance to ICIs^4^.With the purpose of evaluating the response of patients with COAD to ICIs therapy, this study predicted the probability of the NETs-risk model with the use of TIDE, T cell exclusion and dysfunction scores. Relative to the TMB or PD-L1 expression, the TIDE score attains higher predictive accuracies regarding the anti-CTLA4 and -PD-1 therapeutic efficacies, which is a novel approach to forecasting the immunotherapy response^51^. The higher the TIDE score, the worse the tumor response to treatment with ICIs and, accordingly, the inferior the prognosis^52^. The high NETs risk population in this study showed higher TIDE scores, more exclusion and dysfunction of T cells compared to the low NETs risk group. COAD is a heterogeneous disease with diverse genetic backgrounds and clinical manifestations.Tumor cells can alter the behavior of neutrophils and their ability to form NETs by influencing their life cycle through the secretion of cytokines such as IL-6, IL-1β, IFN-γ, and TNF-α.The results showed that COAD patients with either high or low NET-scores manifested respective somatic mutations, including APC,TP53, TTN,MUC16, and KRAS (Fig. 3A,B).Which suggested that COAD might have different interactions with the immune microenvironment depending on the specific genetic alterations and pathways involved.Additionally, M0, M1, and Treg infiltration, as well as the corresponding immune checkpoint molecules CD274, CTLA4, LAG3, PDCD1, TIGI, PDCD1LG2, and HAVCR2, were significantly up-regulated in the high NETs risk group. Also demonstrated by the results, there were positive linkages of NETs to the Treg infiltration, which was further confirmed by multiple immunofluorescence histochemistry and spatial analysis. To sum up, a credible risk signature associated with immunity was developed herein, which enables forecasting of ICI therapy response and survival for COAD patients.

Recent years have witnessed a growing quantity of studies exploring how inflammation affects the carcinogenesis, evolution, and prognosis of COAD. According to the latest evidence, the NETosis has been identified as a viable mechanism of such immunotherapy resistance^18,19, 53^. In pancreatic ductal adenocarcinoma, NETs attenuated tumor response to immunotherapy by preventing CD8 + T cells from contacting carcinoma cells ^19^. Our results further confirm that NETs affected the immune microenvironment, which may be involved in regulating the proportion of CD8T and Treg.Furthermore, although NETs have been reported to restrict the checkpoint blockade immunotherapy response, their pharmacological targeting was accompanied by immunotherapeutic efficacy restoration^18^. As implied by this data, exploring the combinatorial application of NET-targeting therapeutics is necessary for patients with a possible weak response to immunotherapy.MPO, a functional and activation marker of neutrophils, serves as an indicator of the functional state and activity of polymorphonuclear neutrophils (PMN). Consequently, a serum MPO-DNA test kit has been developed and deployed for monitoring NETs levels. The use of drugs such as DNA enzyme (DNase) or Peptidylarginine Deiminase 4 (PAD4) inhibitors, and NE inhibitors can prevent the formation of NETs, thereby eliminating their role in promoting tumor metastasis.This study also provides evidence of an association between inflammation and ICIs-induced therapeutic response.

However, this study still has several limitations that should be dealt with in future research. Firstly, the study constructs and validates a NETs-related risk signature, external validation in independent cohorts would strengthen the robustness of the findings,and the reliance on a single retrospective cohort for validation could potentially cause overfitting, thus constraining the risk signature's generalizability.Prospective study should be carried out further to identify its clinical value via large-cohort and multicenter studies.Secondly, the present work only evaluated the positional relationship between MPO and CD8T,Treg in the TME in terms of spatial position, but the specific interaction mechanism involved is unclear and requires further experimental validation.Thirdly,detailed experimental studies must be added to explore the possible mechanisms of NETs regulate the immune status in TME additional investigations, both in vitro and in vivo, are required.

In conclusion, our study comprehensively elucidated the intricate interplay between NETs modification patterns and the TME, clinical characteristics, and prognosis of COAD. Additionally, we evaluated the predictive value of NETs for treatment sensitivity in ICIs and targeted therapies. Furthermore, we developed a NETs-score system to quantify the NETs patterns in COAD patients and validated the relationship between core gene expression and immune infiltration. Thus, the findings of the present study might facilitate our understanding of COAD and provide ideal strategies for individual treatment.

### Supplementary Information

Supplementary Table 1.

## Data Availability

RNA sequencing data can be accessed via the TCGA and GEO repository. (TCGA) (https://portal.gdc.cancer.gov/), (GEO; accession No.: GSE39582; https://ncbi.nlm.nih.gov/geo/query/acc.cgi?acc=GSE39582). The clinic data of 66 COAD patients at Sun Yat-sen University Cancer Center (SYSUCC) are upload in Table 1.
