# Supplementary material for: A neutrophil extracellular traps-related classification predicts prognosis and response to immunotherapy in colon cancer
Source: Sci Rep. 2023 Nov 7;13:19297. doi: 10.1038/s41598-023-45558-6 (PMC10630512; doi:10.1038/s41598-023-45558-6)
Supplement: Supplementary file 1 — Supplementary Table 1. [file 41598_2023_45558_MOESM1_ESM.docx]

**Supplementary table 1.** The expression patterns of NETs-related genes in normal and COAD samples.

| gene | conMean | treatMean | logFC | pValue | change |
| --- | --- | --- | --- | --- | --- |
| MGAM | 10.37624878 | 0.395754968 | -4.712533703 | 1.18E-11 | DOWN |
| ELANE | 2.282570732 | 0.224788372 | -3.344020252 | 2.87E-26 | DOWN |
| CTSG | 17.92434878 | 2.329853488 | -2.943609565 | 6.94E-23 | DOWN |
| KCNN3 | 3.338429268 | 0.871738266 | -1.937202528 | 4.56E-18 | DOWN |
| TLR7 | 3.599243902 | 1.346020507 | -1.418993479 | 4.18E-16 | DOWN |
| SLC22A4 | 6.39674878 | 2.680259197 | -1.254966301 | 1.74E-07 | DOWN |
| BST1 | 9.073319512 | 4.976364271 | -0.866538369 | 6.26E-11 | DOWN |
| CEACAM3 | 1.874746341 | 1.071387104 | -0.807215573 | 1.07E-09 | DOWN |
| MAPK3 | 206.7760659 | 119.4598636 | -0.791543225 | 1.96E-17 | DOWN |
| HPSE | 12.60633171 | 8.061373996 | -0.645050869 | 4.98E-09 | DOWN |
| SELP | 5.805482927 | 3.795487526 | -0.613130872 | 1.41E-07 | DOWN |
| RIPK3 | 23.93470976 | 15.93047125 | -0.587315367 | 5.37E-11 | DOWN |
| TLR8 | 2.976641463 | 2.038588161 | -0.54611511 | 1.60E-06 | DOWN |
| CRISPLD2 | 22.8927439 | 17.46568541 | -0.390367133 | 0.000732289 | DOWN |
| RIPK1 | 18.35762683 | 14.12176089 | -0.378459573 | 3.50E-11 | DOWN |
| SIGLEC14 | 3.410853659 | 2.678886047 | -0.348499644 | 0.008487501 | DOWN |
| SELPLG | 21.5616439 | 17.33957294 | -0.31439881 | 0.00012593 | DOWN |
| CYBB | 36.08182195 | 31.18534271 | -0.210404076 | 0.000238551 | DOWN |
| ENTPD4 | 23.78414878 | 21.24127125 | -0.163130282 | 0.001979464 | DOWN |
| TECPR2 | 8.83115122 | 7.930011205 | -0.155278614 | 0.03355258 | DOWN |
| ITGAM | 6.444285366 | 6.325022199 | -0.026949838 | 0.001097374 | DOWN |
| ALPL | 5.455526829 | 5.560437209 | 0.027479803 | 0.042855899 | UP |
| ITGB2 | 31.24899756 | 32.20815159 | 0.043615957 | 0.012877725 | UP |
| MME | 7.000780488 | 7.353961522 | 0.071005857 | 1.49E-12 | UP |
| SIGLEC5 | 0.107282927 | 0.120377801 | 0.166148869 | 0.030968193 | UP |
| AKT1 | 29.51499024 | 35.23081205 | 0.255389864 | 0.001189964 | UP |
| ATG7 | 7.86185122 | 9.479245032 | 0.269903099 | 0.000591918 | UP |
| IRAK4 | 11.54210488 | 15.39639133 | 0.415685901 | 3.90E-07 | UP |
| DNASE1 | 4.061295122 | 5.476583932 | 0.431336413 | 1.77E-11 | UP |
| AKT2 | 16.69356829 | 23.47354397 | 0.491743305 | 4.81E-11 | UP |
| SLC25A37 | 11.87241463 | 16.82836047 | 0.503281243 | 0.000604004 | UP |
| F3 | 36.92984878 | 60.61204736 | 0.71481722 | 0.017274724 | UP |
| CREB5 | 1.06032439 | 1.869480973 | 0.818132085 | 0.03452392 | UP |
| HMGB1 | 72.05746341 | 134.451593 | 0.899867077 | 1.07E-11 | UP |
| TLR2 | 2.876885366 | 5.534788795 | 0.94402053 | 0.004715694 | UP |
| FPR1 | 4.892943902 | 9.92387611 | 1.020200982 | 0.025415924 | UP |
| G0S2 | 29.76952439 | 60.48988203 | 1.022857674 | 0.000771155 | UP |
| IL6 | 3.047695122 | 6.390044186 | 1.068107318 | 0.00015785 | UP |
| MPO | 0.148231707 | 0.350274207 | 1.240630679 | 0.000525707 | UP |
| TNFRSF10C | 0.816878049 | 2.039053277 | 1.31970685 | 0.006824807 | UP |
| CYP4F3 | 4.411917073 | 12.49622135 | 1.502014335 | 1.64E-09 | UP |
| FPR2 | 0.770392683 | 2.266492812 | 1.55679568 | 0.043078836 | UP |
| MMP9 | 19.73053415 | 65.51517526 | 1.731399103 | 1.82E-06 | UP |
| IL1B | 9.098914634 | 33.02638372 | 1.859852639 | 7.08E-08 | UP |
| CSF3 | 1.258280488 | 4.672126216 | 1.892625694 | 1.82E-08 | UP |
| FCAR | 0.17195122 | 0.677738901 | 1.978730233 | 0.00035445 | UP |
| CLEC6A | 0.081107317 | 0.331032135 | 2.029067297 | 0.000360135 | UP |
| VNN3 | 0.056892683 | 0.308986681 | 2.441229628 | 1.63E-07 | UP |
| KCNJ15 | 0.124663415 | 0.734938901 | 2.559586181 | 2.40E-09 | UP |
| IL17A | 0.109131707 | 0.685784144 | 2.651684222 | 1.21E-06 | UP |
